# Supplementary material for: Persistent TLR4 Activation Promotes Hepatocellular Carcinoma Growth through Positive Feedback Regulation by LIN28A/Let-7g miRNA
Source: Int J Mol Sci. 2022 Jul 29;23(15):8419. doi: 10.3390/ijms23158419 (PMC9369227; doi:10.3390/ijms23158419)

Supplementary Materials

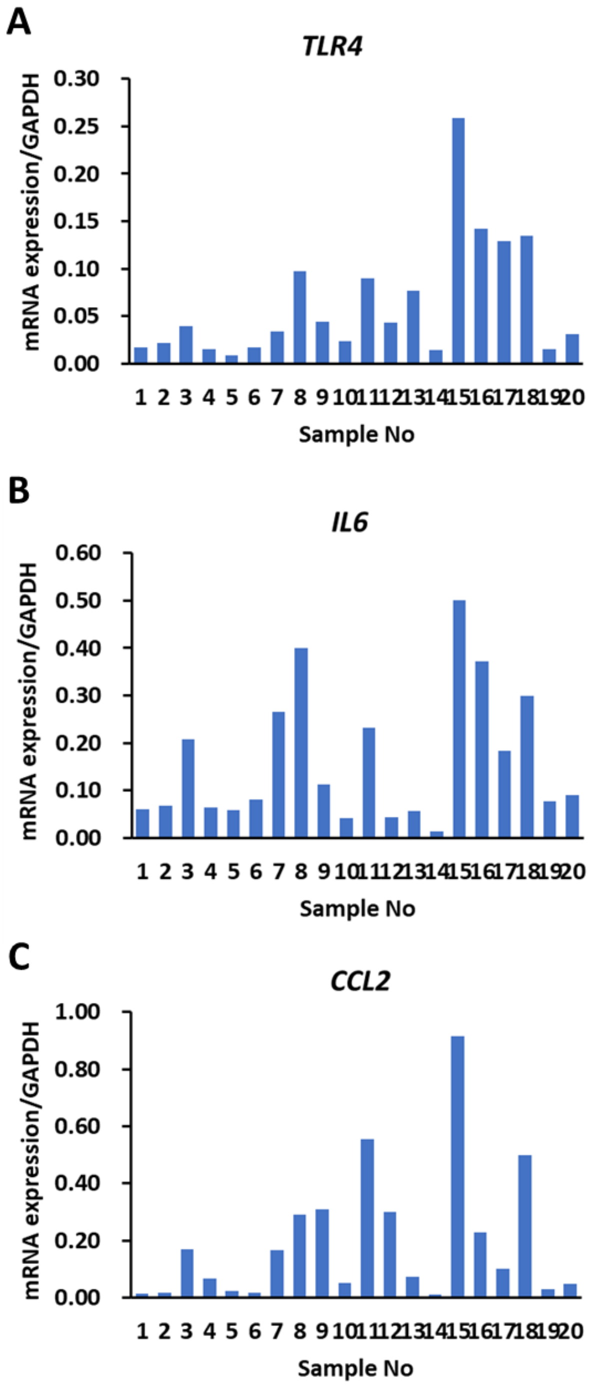

Supplementary Figure S1. mRNA expression of *TLR4*, *IL6* and *CCL2* mRNA in HCC clinical samples. (A) *TLR4*, (B) *IL6*, and (C) *CCL2* mRNA expression level in HCC tumor tissue from 20 patients. The mRNA levels were detected with q-PCR, and results are mean for duplicated measurements.

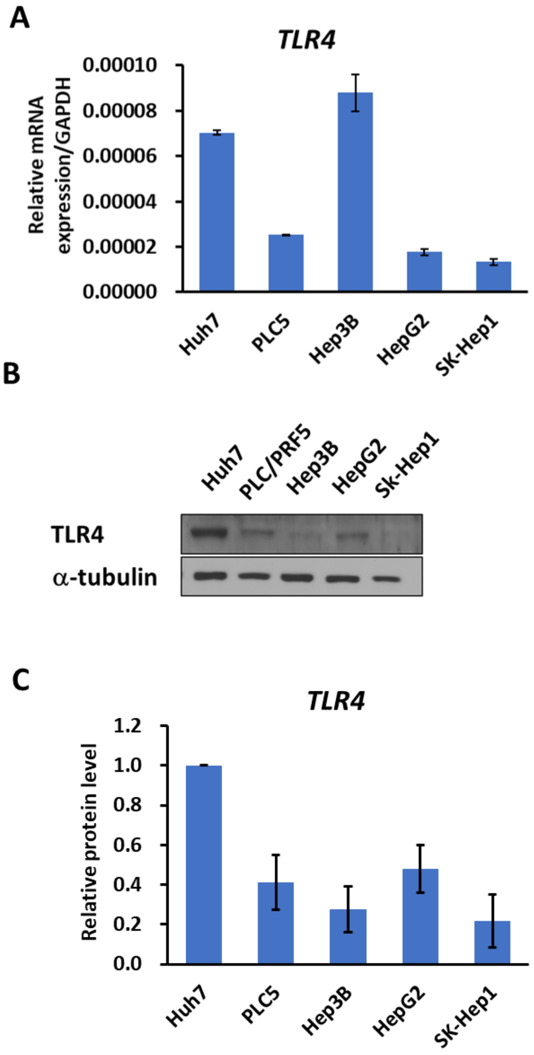

Supplementary Figure S2. TLR4 expression in different human HCC cell lines. (A) mRNA expression level in various human HCC cell lines, including Huh7, PLC5, Hep3B, HepG2 and SK-Hep1 were detected. The mRNA levels were detected with q-PCR. Results of q-PCR are averages ± SD of three separate experiments. (B) Protein expression level in various human HCC cell lines, including Huh7, PLC5, Hep3B, HepG2 and SK-Hep1 were detected. The protein levels were detected with immunoblotting. (C) The quantification of TLR4 protein levels from (B). Error bars show standard deviation.

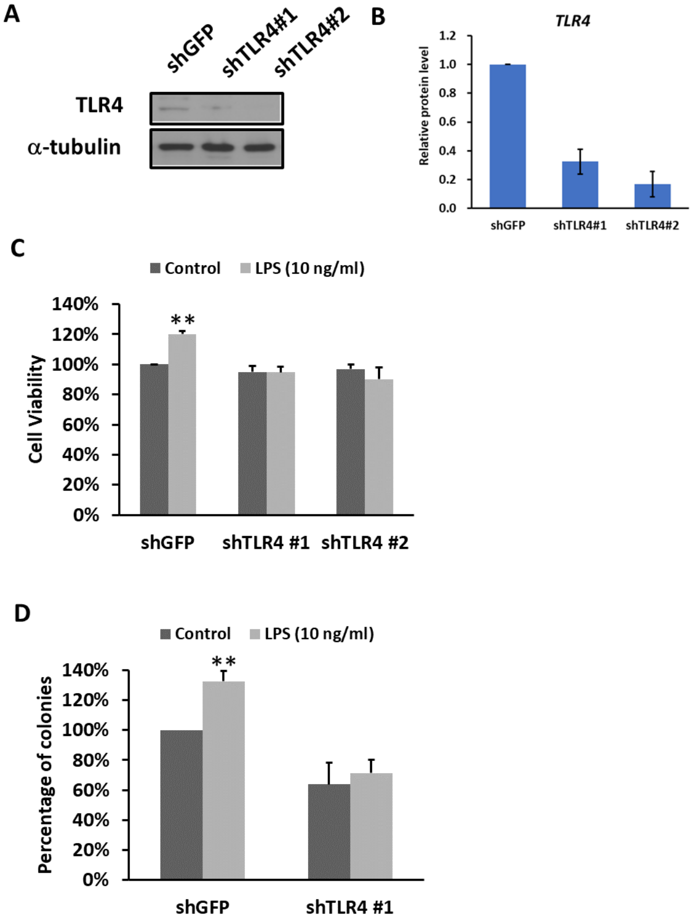

Supplementary Figure S3. TLR4-dependent survival advantage in HCC cell lines. (A) Protein expression level in Huh7 cells with or without shRNA knocked down were detected. The protein levels were detected with immunoblotting. (B) The quantification of TLR4 protein levels from (A). Error bars show standard deviation. (C) Cell viability of Huh7 cells with or without TLR4 silencing in response to LPS. Huh7 cells were plated in 96-well plates and incubated for 5 days with or without LPS stimulation. The cell viability was measured with MTT assays. (D) The clonogenic ability of Huh7 cells with or without TLR4 knocking-down in response to LPS. Huh7 cells were plated in 6-well plates and incubated for 14 days with or without LPS stimulation. The clonogenic ability was determined with colony formation assay.

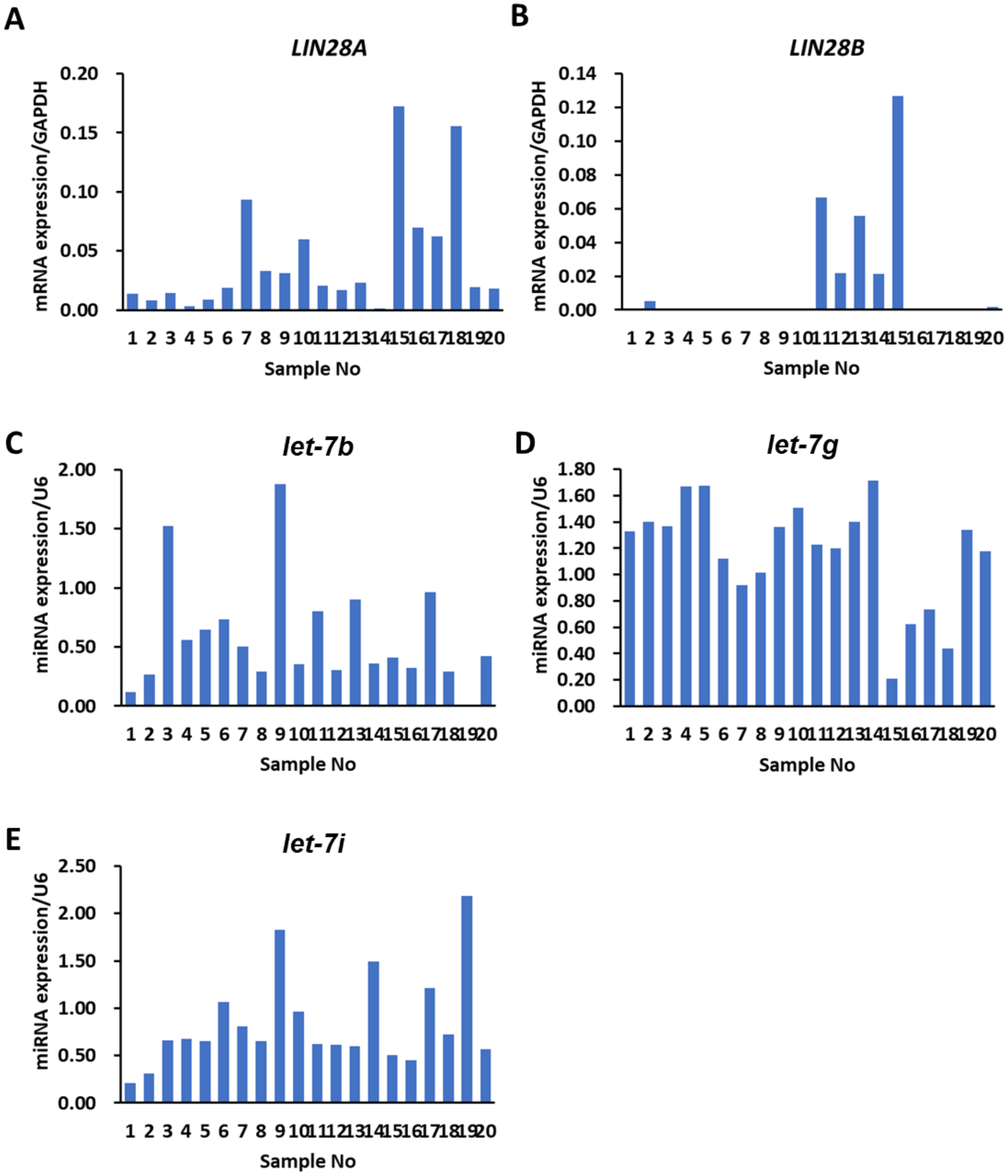

Supplementary Figure S4. Expression level of *LIN28A* and *LIN28B* mRNA, and *let-7b*, *let-7g* and *let-7i* miRNA in clinical HCC samples. (A) *LIN28A* and (B) *LIN28B* mRNA, and (C) *let-7b*, (D) *let-7g* and (E) *let-7i* miRNA expression level in tumor tissues of patients with HCC. The mRNA and miRNA levels were detected with qPCR, and results are mean for duplicated measurements.

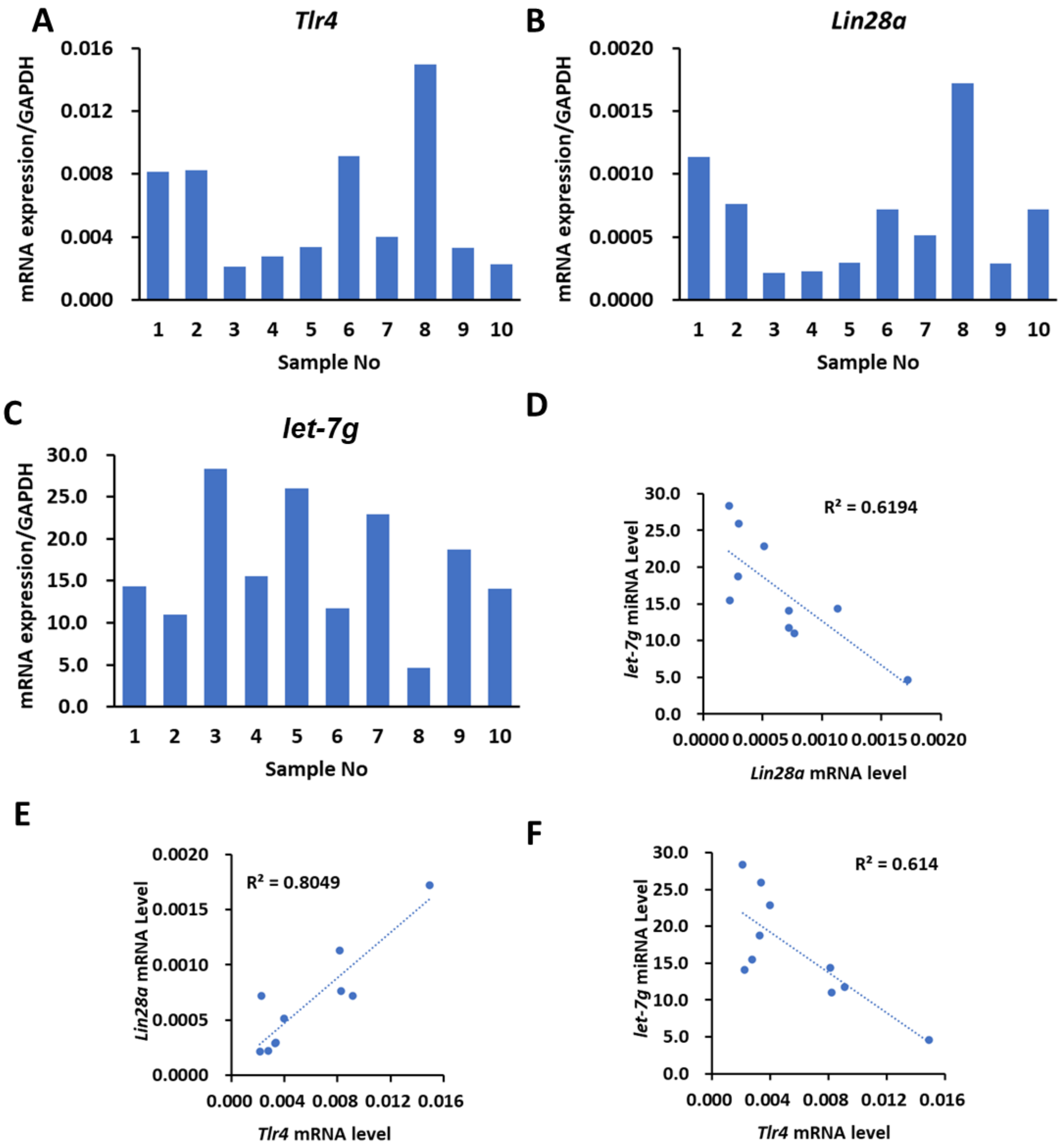

Supplementary Figure S5. The expression of Tlr4, Lin28a and let-7g in mouse HCC cells. (A) *Tlr4* mRNA level and (B) *Lin28a* mRNA level, and (C) *let-7g* miRNA level in mouse HCC cells. Fifteen-day-old BL/6 males were given DEN. After 8 months, HCC cells were isolated from DEN-induced tumors. qPCR analysis was used to determine mRNA or microRNA level. (D, E, F) Correlation between the expression of *Tlr4* and *Lin28a* mRNA and *let-7g* miRNA in HCC patients. Pearson's correlation analyses were performed. Data are presented as each value. (R^2, correlation coefficient).

Supplementary Table S1. Primers' sequence used in Q-PCR.

Gene	Primer Sequence
Human *TLR4*	F : 5'- TGAGCAGTCGTCTGGTATC -3'<br>R : 5'- CAGGGCTTTTCTGAGTCGTC -3'
Human *IL-6*	F : 5'- AAATGCCAGCCTGCTGACGAAG -3'<br>R : 5'- AACAACAATCTGAGGTGCCCATGCTAC -3'
Human *CCL2*	F: 5'- CAGCCAGATGCAATCAATGCC -3'<br>R: 5'- TGGAATCCTGAACCCACTTCT -3'
Human *LIN28A*	F : 5'- CAAAAGGAAAGAGCATGCAGAAG -3'<br>R : 5'- GCATGATGATCTAGACCTCCACA -3'
Human *LIN28B*	F : 5'- GCCCCTTGGATATTCCAGTC -3'<br>R : 5'- TGACTCAAGGCCTTTGGAAG -3'
Human *GAPDH*	F : 5'- GTCATCATATTTGGCAGGTT -3'<br>R : 5'- GAAGGACTCATGACCACAGT -3'
Human *c-MYC*	F : 5'- AATGAAAAGGCCCCCAAGGTAGTTATC -3'<br>R : 5'- GTCGTTTCCGCAACAAGTCCTCTTC -3'
Mouse *Tlr4*	F : 5'- GCTTTCACCTCTGCCTTCAC -3'<br>R : 5'- CGAGGCTTTTCCATCCAATA -3'
Mouse *Lin28a*	F : 5'- CAAAAGGAAAGAGCATGCAGAAG -3'<br>R : 5'- GCATGATGATCTAGACCTCCACA -3'
Mouse *Lin28b*	F : 5'- CTGATGGATCAGATGTGGACTG –3'<br>R : 5'- TCTTTGGCTGAGGTAGA -3'
Mouse *Cph*	F : 5'-ATGGTCAACCCCACCGTGT-3'<br>R : 5'-TTCTTGCTGTCTTTGGAACTTTGTC-3'
Human *let-7b*	F : 5'- GGAGTGAGGTAGTAGGTTGT -3'
Human *let-7g*	F : 5'- GGAGTGAGGTAGTAGTTTGT -3'
Human *let-7i*	F : 5'- GGAGTGAGGTAGTAGTTTGT -3'
Human *U6*	F : 5'- CTCGCTTCGGCAGCAC -3'<br>R : 5'- AACGCTTCACGAATTTGCG -3'
Mouse *let-7b*	F : 5'- GGAGTGAGGTAGTAGGTTGT -3'
Mouse *let-7g*	F : 5'- GGAGTGAGGTAGTAGTTTGT -3'
Mouse *let-7i*	F : 5'- GGAGTGAGGTAGTAGTTTGT -3'
Mouse *U6*	F: 5'- CTCGCTTCGGCAGCACA -3'<br>R: 5'- AACGCTTCACGAATTTGCGT -3'
Stem-loop	R : 5'- GTGCAGGGTCCGAGGT -3'

Figure S2D

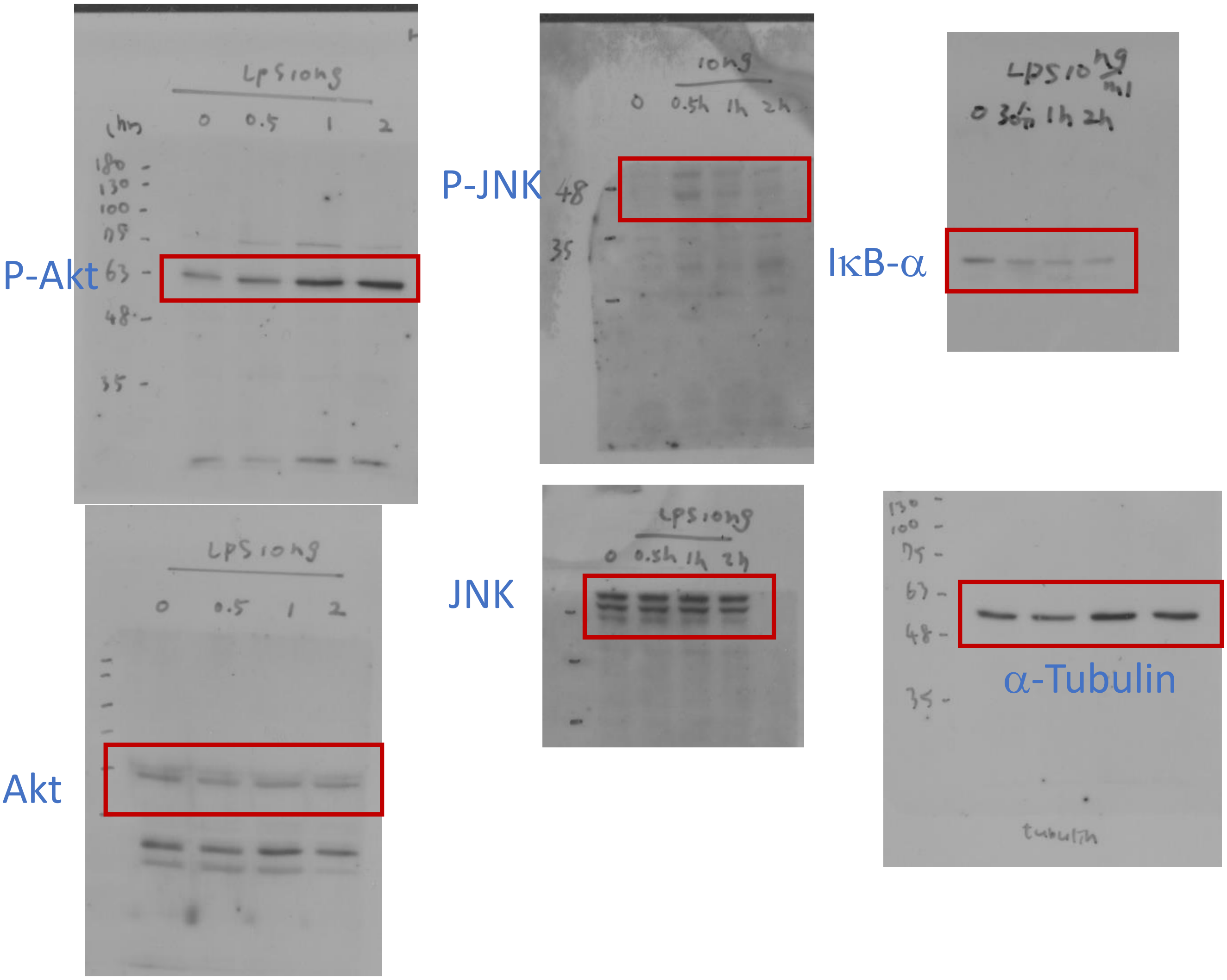

Figure S2E

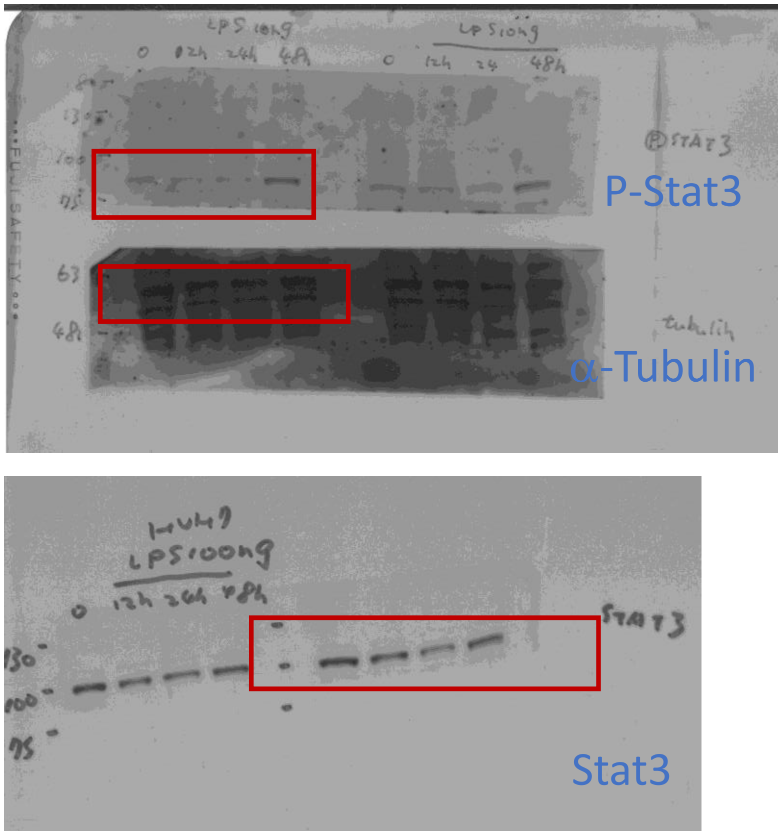

Figure S5C

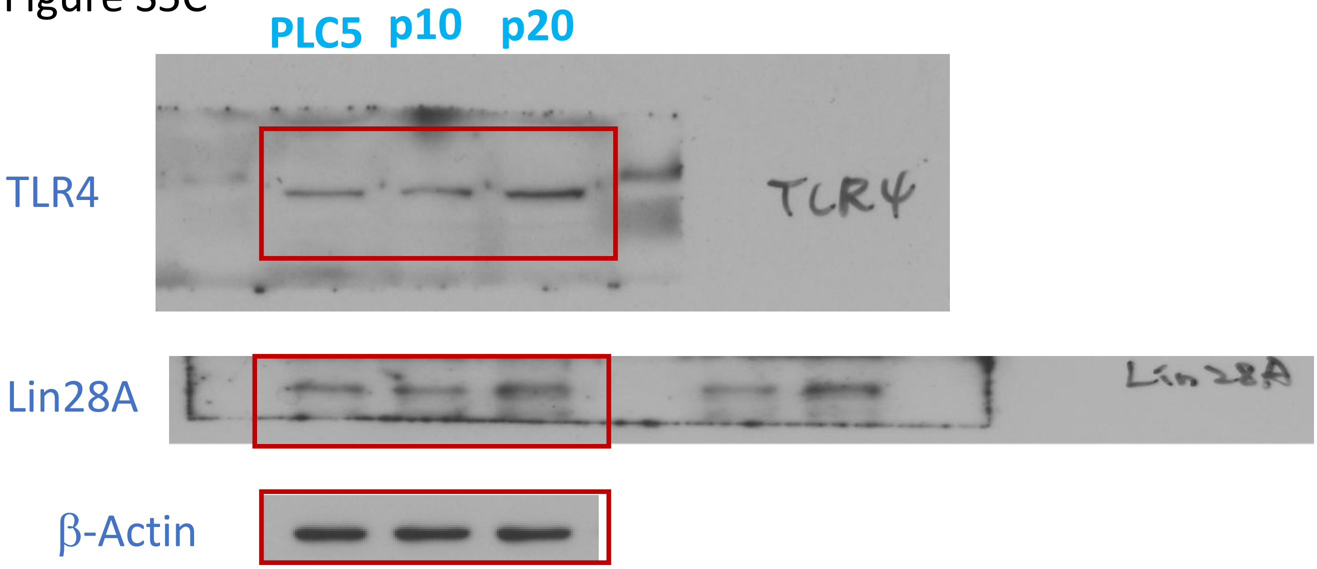

Figure S5G

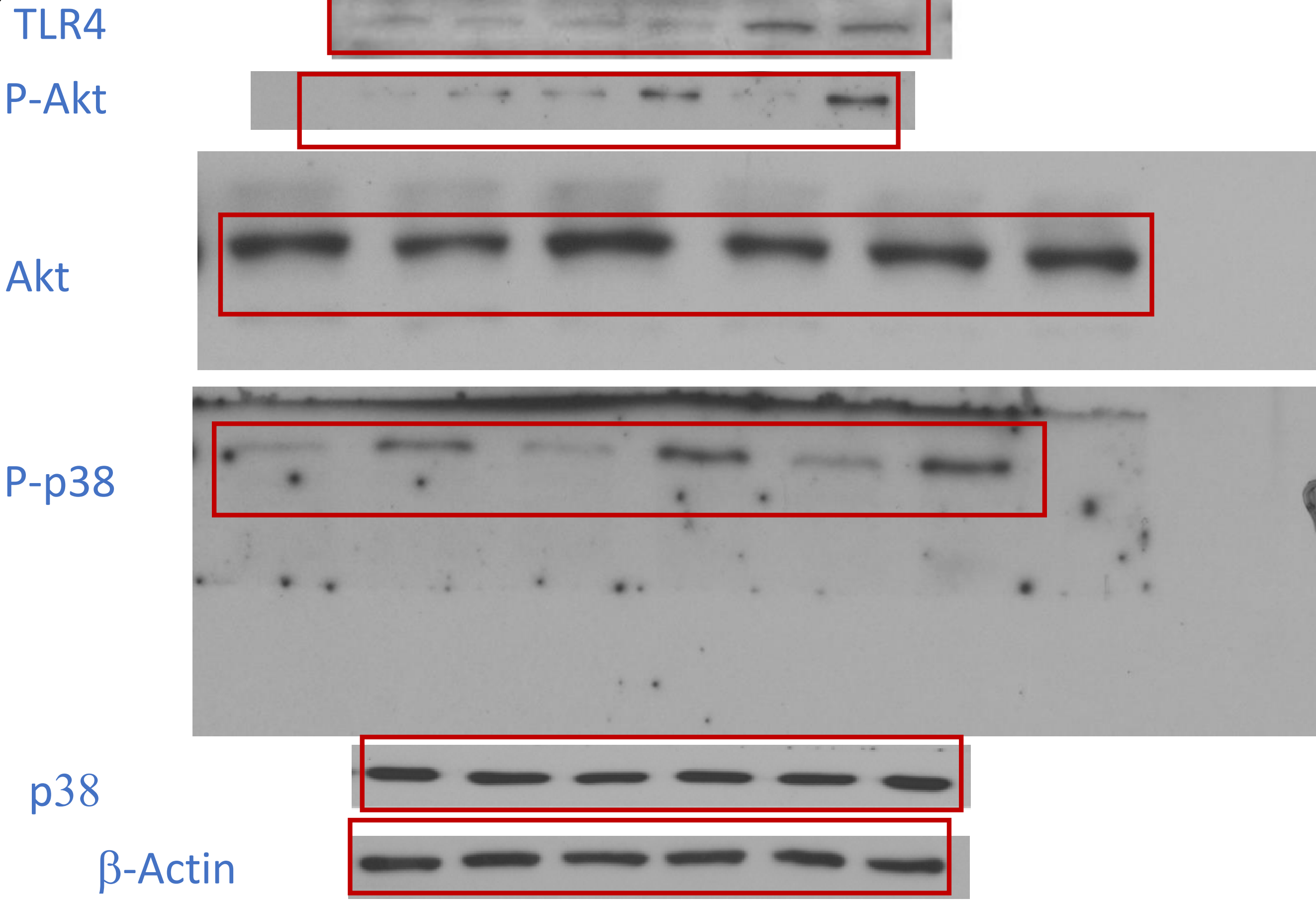

Figure S7D

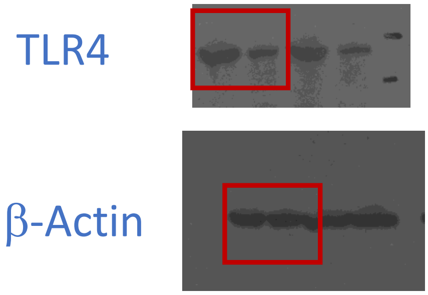

Supplementary Figure S2 B

TLR4

α-Tubulin

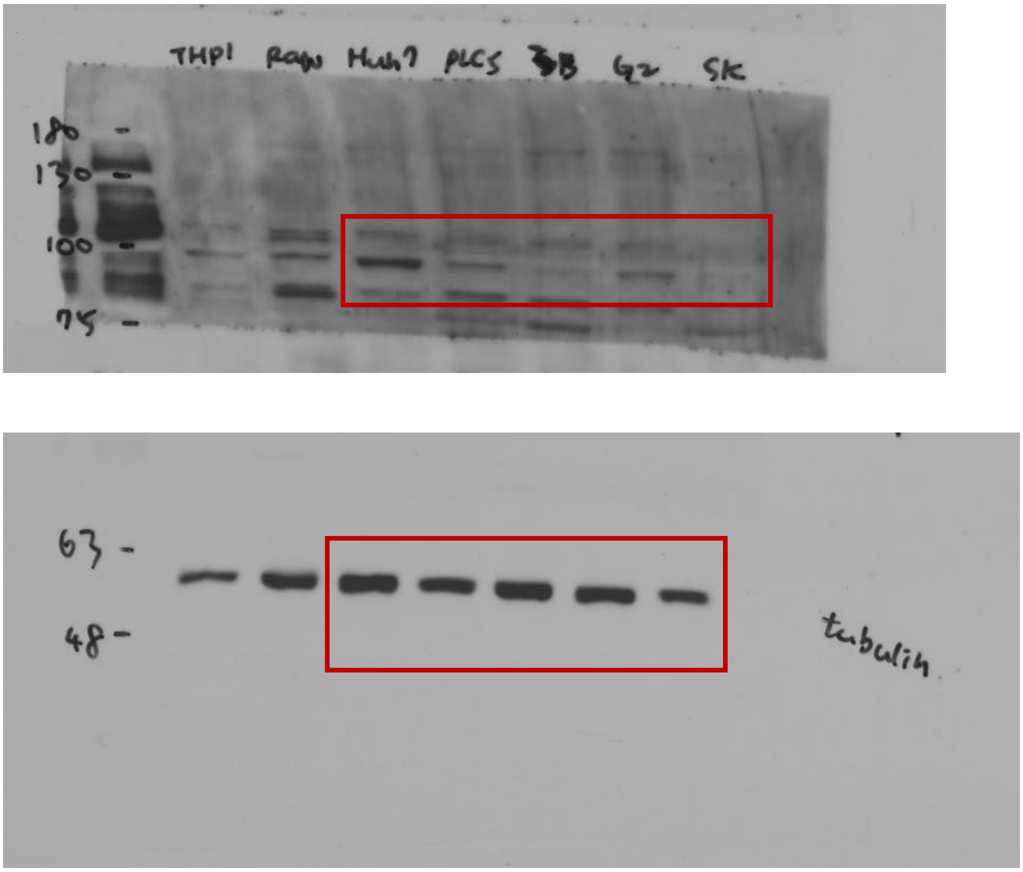

Supplementary Figure S3 A

TLR4

α-Tubulin

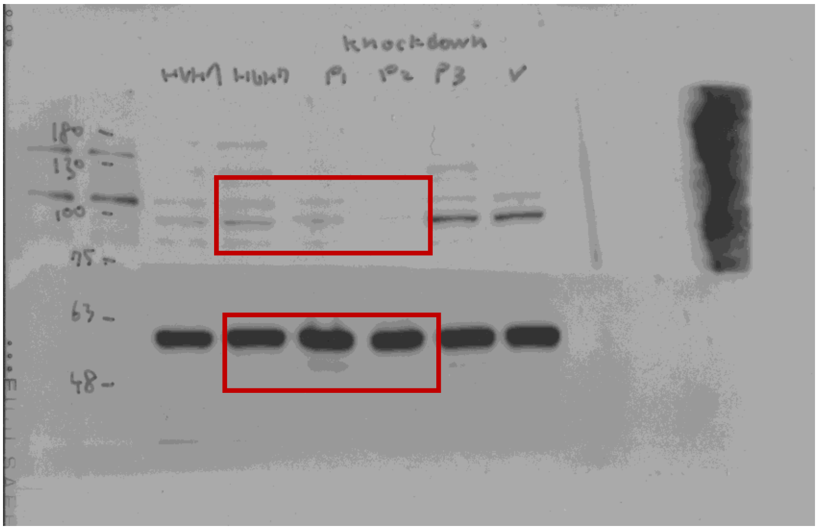

Supplement: Supplementary file 1 [file ijms-23-08419-s001.zip › ijms-1699367-supplementary.pdf]
